# Psychometric properties of a new self-report measure of medical student stress using classic and modern test theory approaches

**DOI:** 10.1186/s12955-020-01637-0

**Published:** 2021-01-02

**Authors:** Matthew J. Mosquera, Aaron Kaat, Melinda Ring, Gaurava Agarwal, Sydney Glickson, David Victorson

**Affiliations:** 1 Department of Psychiatry, Brigham and Women’s Hospital, Harvard Medical School, 60 Fenwood Avenue, Boston, MA 02115 USA; 2 Department of Medical Social Sciences, Northwestern University Feinberg School of Medicine, Chicago, IL USA; 3 Osher Center for Integrative Medicine, Northwestern University Feinberg School of Medicine, Chicago, IL USA; 4 Department of Medical Education and Department of Psychiatry and Behavioral Sciences, Northwestern University Feinberg School of Medicine, Chicago, IL USA

**Keywords:** Stress, Medical student, Measurement, Item response theory

## Abstract

**Background:**

Medical students face significant stressors related to the intense rigors of their training and education. Accurate measurement of their stress is important to quickly identify, characterize and ameliorate these challenges. Existing measures have limitations that modern measurement approaches, such as item response theory (IRT), are able to address. This study presents the calibration and validation of a new IRT-based measure called the Medical Student Stress Scale (MSSS).

**Methods:**

Following rigorous measurement development procedures described elsewhere, the authors created and tested a pool of 35 items with 348 1st – 4th year medical students along with demographic and external validity measures. Psychometric analysis included exploratory and confirmatory factor analyses, IRT modeling, and correlations with legacy measures.

**Results:**

Of the original 35 items, 22 were retained based on their ability to discriminate, provide meaningful information, and perform well against legacy measures. The MSSS differentiated stress scores between male and female students, as well as between year in school.

**Conclusion:**

Developed with input from medical students, the MSSS represents a student-centered measurement tool that provides precise, relevant information about stress and holds potential for screening and outcomes-related applications.

## Background

It is widely understood that medical school can be a very stressful experience that is different from other forms of life stress. This includes exposure to death and human suffering, ethical conflicts, adjustment to the pressures of the medical school environment, student abuse, personal life events, and educational debt [1, 2]. While stress can play an adaptive role in providing that extra motivation and “push” in times of intense study, if left unidentified or unmanaged, stress may manifest in detrimental ways such as impaired sleep and appetite, symptoms of depression and anxiety, and at worst, suicide [3–5]. This may result in downstream consequences such as poor academic performance, cynicism, academic dishonesty, and/or substance abuse. Heightened stress contributes to roughly 25% of medical students considering dropping out [4]. What’s more is that only roughly 16% of medical students who screen positive for depression actually seek psychiatric treatment [5]. In order to create sustainable interventions, it is crucial to effectively measure medical student stress in the most precise way possible.

While global measurement tools exist to assess stress or burnout, such as the Perceived Stress Scale (PSS) and Maslach Burnout Inventory (MBI), they are designed for use across broad populations and do not capture the specific experience of medical students. For student populations in general, multiple scales have been created to gauge stress levels; however, each comes with limitations and are not ideal for use within medical student populations. For example, the Student-Life Stress Inventory (SSI) has a significant focus on physical stress responses and universally stressful scenarios, and the combination of stress factors experienced specifically by medical students is not represented by its broader items [6]. Other student stress scales such as the Undergraduate Stress Questionnaire (USQ) and the Scale for Assessing Academic Stress (SAAS) are not designed for the context of medical students [7, 8]. Other metrics, such as the Perception of Academic Stress Scale (PAS), are focused on test anxiety and were developed to test stress levels in courses where grades depended primarily on a singular exam [9]. The stressors of medical school encompass far more than the stress of an individual exam. For medical student stress specifically, three different measures exist, each with its own limitations; Perceived Medical Student Stress (PMSS) Instrument [10], Medical Student Stress Profile (MSSP) [11], and Medical Student Stressor Questionnaire (MSSQ) [12]. The PMSS is a 13-item measure containing several items that can be considered double-barreled (e.g., they measure two or more different things), such as “Medical school is cold, impersonal and needlessly bureaucratic.” Several items are also negatively phrased, which can be cognitively complex to understand when double negatives occur. Finally, it includes certain colloquialisms (e.g., “baptism by fire”) that may not be fully understood by all respondents. The MSSP is a 52-item measure, which, in addition to its length (and associated response burden) also instructs respondents to rate each item twice; once to measure how true the item is, and then to measure how stressful it is. This can create unnecessary cognitive load and response burden as it actually requires a respondent to answer 102 items. Finally, the MSSQ is either a 20 or 40 British English item measure that contains a list of possible stressors, versus items that are written in a more common question or statement form (e.g., “heavy workload”, “large amount of content to be learnt”, “falling behind in reading schedule”). A list of issues is not necessarily a limitation in and of itself, however, given the nature of medical student stress, a respondent may more easily and quickly identify with an item’s content and meaning when it is written in a more personal way.

Given these shortcomings, the purpose of this current study was to use established measurement development methodologies based on the Patient Reported Outcomes Measurement Information System® (PROMIS) [13] to develop and test a new measure of medical student stress.

## Methods

### Overview

This study was approved by the participating institution’s internal review board. The development and testing of this new measure drew from a widely accepted multi-step, multi-phase measurement development methodology based on the PROMIS methodology, which included the following: PHASE I: 1) A literature search of existing measures, concepts, and items; 2) Development of a guiding conceptual framework; 3) Medical student group discussions to elucidate and confirm important concepts and issues related to medical student stress; 4) Creation of an initial pool of medical student stress items; 5) Refinement of items via expert review; 6) Cognitive interviews with medical students; and 7) Final expert item review; PHASE II: The final item pool was administered to a sample of actively enrolled medical students attending a large, private Midwestern university, with an average class size of 160 students (total number of enrolled students was roughly 640 students). Given that PHASE I activities have been previously reported [14] this report will focus exclusively on PHASE II activities. Overall study flow is graphically represented in the supplementary online material (Additional file 1: Figure-SF1).

### Calibration testing procedures

Eligible participants were current medical students (1st - 4th year also known as “M1-M4”) at the participating institution. Inclusion criteria for participants included students in either the MD or MD/PhD programs actively enrolled in coursework and/or clinical rotations. Exclusion criteria included MD/PhD students in the research portion of their program. There were no power calculations used to determine sample size. We attempted to enroll > 200 eligible participants who represented the target population. Following informed consent, the authors administered a 10–15 min online survey via Research Electronic Data Capture (REDCap), which is a secure web-based data collection application. Participation was voluntary and responses were kept anonymous. Secure invitations were sent via an encrypted email service, and survey participants were notified that taking part in the survey would have no bearing on academic standing whatsoever.

### Measures

In addition to new items of medical student stress, the authors also administered the following socio-demographic form and legacy measures to establish preliminary convergent validity evidence: 1) *Socio-Demographic Form*: This included year in medical school, gender, race (either Caucasian or non-Caucasian), and religious belief; 2) *Health and Lifestyle Behaviors*. Using single-item statements with a 5-point Likert response scale, the authors asked participants about their sleep and exercise patterns, including questions on frequency of exercise, average total hour of sleep per night, and impact from sleep and exercise on stress; 3) *Burnout.* To measure burnout, the authors administered the 10-item Burnout Measure Short Version [15]; 4) *Perceived Stress****.*** To gauge stress levels, the authors used the Perceived Stress Scale-4 (PSS-4) [16]; 5) *Anxiety*. To assess anxiety, the authors used the 4-item PROMIS Anxiety Short Form, which was drawn from a 29-item bank [17]; 6) *Visual Analog Scale (VAS).* To assess current stress levels, the authors used a VAS in the form of a single question which asked participants to rate their perceived stress levels on a 10-point Likert-type scale ranging from no stress at all (1) to worst stress imaginable (10). See Table 1 for the validity measure characteristics (domains, number of items, and reliabilities) used in this study.

**Table 1:** Validity measure characteristics

	Dimensions	Number of items	Score range	Reliability (Cronbach’s *α*)
Burnout Measure [15]	Emotional Exhaustion	10	1–7	0.85–0.87
PROMIS Anxiety-4 [17, 22]	Fear, anxious misery, hyperarousal, and somatic symptoms related to arousal	4	4–20	0.93
Perceived Stress-4 [23]	Perceived Stress	4	0–16	0.77
Visual Analog Scale	Current Stress Level	1	1–10	n/a

### Analysis

Following data cleaning, the authors first conducted a series of exploratory item factor analyses (EFAs), including unidimensional, 2- and 3-Factor solutions. Consistent with previous studies, we used a combination of statistical factor enumeration strategies and theory [18]. The number of factors to extract was guided by the theoretical model utilized when developing the questionnaire. Targeted EFA rotations allowed the authors to explore these theoretical models of stress and burnout (e.g. reasons, reactions, and responses to stress). The optimal model was chosen based off of a combination of model fit (utilizing the Akaike and Bayesian Information Criteria [AIC and BIC, respectively], and Velicer’s minimum average partial [MAP] test, all of which favor models with lower values) and interpretability of the factor solution.

The optimal model was then fit using a confirmatory item factor analysis (CFA), and used for reliability and validity evaluations. If significant differences emerged between the EFA and CFA results, further item and scale refinement occurred. Then, we used item response theory (IRT) for scoring. The final items were calibrated using the graded response model [19]. Given that our theoretical model suggested potential multidimensionality, we did not restrict the GRM to the unidimensional case but would allow multidimensional IRT if indicated from the EFA and CFA results. Other IRT assumptions were also evaluated, including visual inspection of responses of non-parametric response curves for monotonicity and inspection of specific-factor loadings and residuals from the CFA results to examine local dependence.

Cronbach’s alpha and McDonald’s omega was used to index internal consistency reliability, and Pearson correlation coefficients with external measures were used to index validity. T-scores derived from the optimal model were also used in known group’s discriminant validity t-test evaluations. The authors hypothesized that the optimal model for the MSSS would exhibit high internal consistency (α > 0.80), and correlate moderately with validity measures (r > 0.50) [20]. The authors also hypothesized that there would be at least a small observed effect size difference (d > 0.20) between known groups.

## Results

Following all item development activities, the authors arrived at a field testing-ready item pool of 35 items. The item context for all items is “Since starting medical school” with response options: 0 = Never, 1 = Rarely, 2 = Sometimes, 3 = Often, and 4 = Always.

### Calibration testing results

In total, 348 medical students completed the survey (M1 = 144 (41%), M2 = 145 (42%), M3 = 26 (7%), M4 = 33 (9%)), of which 175 (50%) were male and 173 (50%) were female. In terms of ethnicity, 197 (57%) responded white/Caucasian, 155 (45%) responded non-Caucasian, and 2 (1%) did not respond.

As is typical with factor enumeration, indices did not provide clear support for the same number of factors. The AIC and BIC favored a 3-factor solution (1-factor AIC 95% CI 29733–29,734 BIC 30403–30,405; 2-factor AIC 29284–29,286 BIC 30085–30,087; 3-factor AIC 29124–29,126 BIC 30053–30,055), while the MAP slightly favored the 2-factor solution (1-factor 0.014; 2-factor 0.010; 3-factor 0.011). As the 3-factor model was more consistent with the theoretical model, the authors selected this model and labeled the factors: 1) Social Challenges; 2) High Activation; and 3) Low Activation. The Social Challenges factor represented items such as difficulty asking for help, feeling unsupported by faculty and peers, feeling taken advantage of by faculty, and feeling pressure to get good grades. The High Activation factor represented items such as feeling anxious, being unable to relax, being overly self-critical, and feeling overwhelmed. Finally, the Low Activation factor represented items such as feeling hopeless, depressed, having difficulty motivating oneself, and feeling like dropping out of school. See Table 2 below for item coefficients by factor. Note that generally, item coefficients >.30 characterize each respective factor, however in the case of cross-loadings (due to conceptual overlap), the higher coefficient is to be used.

**Table 2:** Exploratory factor analysis item coefficients

Item ID	Item (each begins with “Since starting medical school”)	Social challenges	High activation	Low activation	Items removed
msss1	I notice fluctuations in appetite.	0.08	0.03	**0.46**	
msss2	I have difficulty asking for help.	**0.45**	0.21	0.2	
msss3	I have trouble falling/staying asleep.	−0.02	0.24	**0.38**	x
msss4	I receive less satisfaction from learning.	0.11	−0.04	**0.65**	
msss5	I am unable to relax.	−0.05	**0.69**	0.23	
msss6	I feel anxious.	−0.08	**0.69**	0.27	
msss7	I am unable to enjoy activities outside of classes/rotations.	0.01	**0.49**	0.29	
msss8	I feel pressure extracurricular activities (student groups, research, etc.).	0.2	**0.37**	−0.11	x
msss9	I notice that I drink alcohol in excess.	0.02	−0.08	**0.33**	x
msss10	I use drugs in excess (prescription and/or non-prescription).	−0.01	− 0.04	**0.47**	x
msss11	I feel hopeless that I will ever get my degree.	0.08	0.25	**0.59**	
msss12	I feel depressed.	0	0.32	**0.61**	
msss13	I am stressed about finances.	0.17	0.07	0.28	x
msss14	I have a hard time motivating myself to study.	0.18	−0.06	**0.62**	
msss15	I feel emotionally exhausted.	0.11	**0.46**	0.38	
msss16	I feel bothered by the amount of exposure to death and human suffering.	**0.3**	0.06	0.17	x
msss17	I am fearful of failing.	0.13	**0.52**	0.28	
msss18	I exercise less.	0.18	0.18	0.15	x
msss19	I feel unsupported by my peers.	**0.54**	0.18	0.09	
msss20	I feel competition from my peers.	**0.54**	0.29	−0.19	
msss21	I feel unsupported by faculty.	**0.8**	−0.09	0.16	
msss22	I feel taken advantage of by faculty (i.e. research mentors, professors, and/or school administrators).	**0.68**	−0.1	0.02	
msss23	It is challenging to maintain relationships with others outside of school.	0.08	**0.34**	0.19	x
msss24	I feel pressure from others (parents, professors, mentors, etc) to get good grades.	**0.39**	0.23	0.06	
msss25	I feel unmotivated to attend class.	0.18	−0.06	**0.41**	x
msss26	I feel pressure from myself to get good grades.	0.1	**0.71**	−0.38	x
msss27	I am overly self-critical.	−0.01	**0.85**	−0.03	
msss28	I feel need to be perfect.	0.2	**0.77**	−0.29	x
msss29	I am unsure of abilities as student.	0.07	**0.49**	0.36	
msss30	I hardly have enough time to get things done.	−0.04	**0.41**	0.32	
msss31	I feel overwhelmed by everything there is to do.	0.01	**0.53**	0.43	
msss32	I struggle maintaining a healthy school-life balance.	0.15	**0.59**	0.16	
msss33	It is challenging to start or maintain romantic relationships.	0.12	**0.31**	0.19	x
msss34	I think about dropping out of school.	0.09	0	**0.79**	
msss35	I question my decision to enter medical school.	0.15	−0.05	**0.76**	x

Coefficients in bold characterize respective factors; correlations between factors High Activation and Social Challenges = .42; correlation between factors Low Activation and Social Challenges = .36; Correlation between factors Low Activation and High Activation = .31. Due to conceptual and psychometric misfit (e.g., model explained less variance/greater error in ratings) we removed items found in the “Items Removed” column. As before, each item stem has the same contextual qualifier – “Since starting medical school”

The authors removed two items that did not load well on any factor (e.g., stress about finances and exercising less), as well as two items with negative cross loadings (e.g., pressure to get good grades and need to be perfect). The authors retained other items with cross-loadings if they were conceptually/clinically relevant for content validity. It is important to note that while there are plausible 3-factors, they are not necessarily conceptually “separate” and one scale could still give a precise score that encompasses all three.

The authors proceeded with testing a 3-factor solution in a restricted, hierarchical CFA model, allowing cross-loading items to co-load between High and Low Activation. The High and Low Activation factors came to represent locally dependent doublets or triplets (e.g., alcohol & drugs: msss9 & msss10; pressure: msss26, msss27, & msss28; dropping out: msss4, msss34, & msss35; and feeling unmotivated: msss14 & msss25), rather than two distinct factors. The authors decided to remove one item in locally dependent pairs and 1–2 items in locally dependent triplets, and remove items with poor remaining relationships, based on content relevance and available item information. The optimal final model was a bi-factor model with a general factor representing stress/burnout and a specific factor with six items (msss2, msss19, msss20, msss21, msss22, msss24) representing both general stress/burnout and social challenges. This is graphically represented in the supplementary online material (Additional file 2: Figure-SF2).

### Item calibration using item-response theory modeling

The retained 22 items underwent item response theory (IRT) bi-factor calibration, which provides specific information about each item’s discriminability and performance along a severity continuum from mild to severe. Marginalizing the social challenges factor to emphasize the stress/burnout, the primary factor provided item slopes (e.g., how discriminating each item is), item thresholds (e.g., how difficult each item is in order for a person to endorse a specific response category), marginal item characteristic curves (e.g., a visual depiction of each item’s discrimination between response categories and how informative an item is across a continuum), and a test information function (e.g., how informative and precise the entire set of items is across the continuum of the latent trait) [18] . The IRT parameters, marginal item characteristic curves, and marginal information plots, are available as online supplementary material (Additional file 3: Table-ST1).

**Fig. 1:**
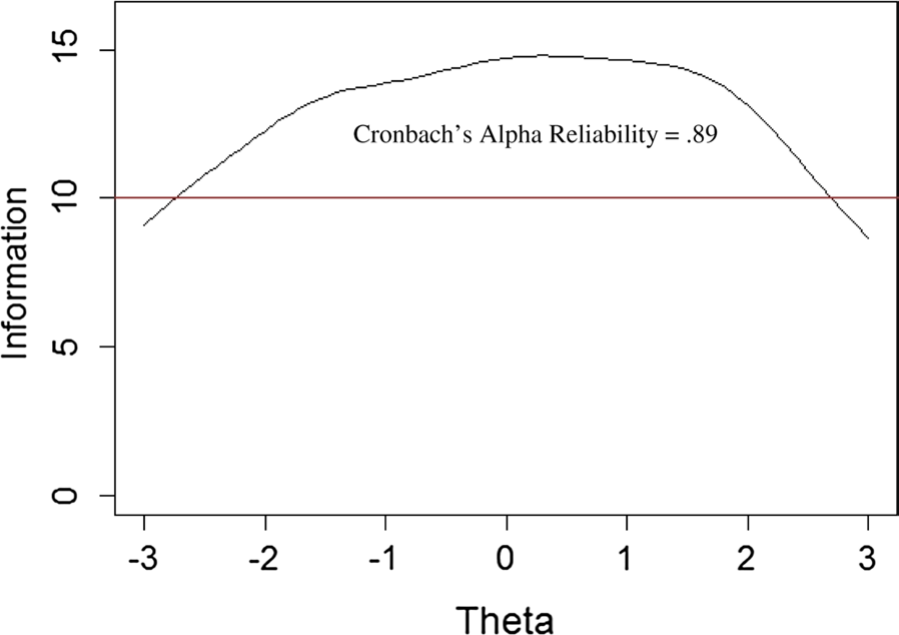
MSSS test information function

Figure 1 below illustrates how well the MSSS estimates a respondents’ latent trait of medical student stress over the whole range of scores. Since test information function will be much higher than any single item information function, a test measures ability more precisely than does a single item. The MSSS is a reliable scale (Cronbach’s α = 0.89; omega_total_ = 0.94; omega_hierarchical_ = 0.91), covers a wide range of medical student stress, and only declines in precision (i.e. reliability) towards the very extremes of stress.

### Administering and scoring the MSSS-22

See Table 5 for MSSS-22 instructions and recall period, response options, and items.

Items from the MSSS-22 may be summed into a total score and converted into a T Score with a mean of 50 and standard deviation of 10 by using the conversion table are available as online supplementary material (Additional file 4: Table-ST2).

### Validity evidence with external validity measures

Convergent validity was established with moderately high associations with the Burnout Scale Short Version (r = 0.800, *p* < .01), PROMIS Anxiety (r = 0.672, *p* < .01), PSS-4 (r = 0.739, *p* < .01), and a stress visual analog scale (r = 0.641, *p* < .01) (see Table 3). Criterion-related validity was established with small inverse associations with self-reported regularity of exercise (r = − 0.261, *p* < .01) and hours of sleep on average (r = − 0.237, *p* < .01).

**Table 3:** Associations between MSSS & validity measures

	MSSS
Burnout measure	.800^a^
PROMIS anxiety-4	.672^a^
Perceived stress-4	.739^a^
Visual analog scale (current stress)	.641^a^
Regularity of exercise	−.261^a^
Hours of sleep on average	−.237^a^

^a^Correlation is significant at the 0.01 level (2-tailed).

Known Groups validity was established with statistically significant differences in the MSSS scores between M1s and M2s, and also between male and female medical students (Table 4). Given the more complete samples for M1s and M2s, known groups validity focused on these cohorts as opposed to M3s and M4s with less complete samples. Other validity measures, such as the burnout measure short version and PSS-4, were unable to significantly differentiate difference between M1s and M2s; however, the Burnout Measure was also able to demonstrate a significant difference between male and female students (Table 4).

**Table 4:** Known-groups validity

	Medical student year			Gender		
	M1 (***n*** = 144)	M2 (***n*** = 145)		Male (***n*** = 174)	Female (***n*** = 173)	
	Mean	Mean	Sig (p)	Mean	Mean	Sig (p)
MSSS	50.95	48.53	0.03	47	52.5	< 0.01
Burnout measure	32.5	30.9	0.151	2.9	3.4	< 0.01
PSS-4	10.5	10.8	0.196	10.6	10.6	0.96

**Table 5:** MSSS scale instructions, recall period, items, and response options

Instructions & recall period:	The questions in this scale ask about your well-being since starting medical school. In each case, please indicate your response.
Response options:	0 = Never, 1 = Rarely, 2 = Sometimes, 3 = Often, 4 = Always
1. Since starting medical school,	I notice fluctuations in my appetite.
2. Since starting medical school,	I have difficulty asking for help.
3. Since starting medical school,	I receive less satisfaction from learning new material.
4. Since starting medical school,	I am unable to relax.
5. Since starting medical school,	I feel anxious.
6. Since starting medical school,	I am unable to enjoy activities outside of classes/rotations.
7. Since starting medical school,	I feel hopeless that I’ll ever get my degree.
8. Since starting medical school,	I feel depressed.
9. Since starting medical school,	I have a hard time motivating myself to study
10. Since starting medical school,	I feel emotionally exhausted.
11. Since starting medical school,	I am fearful of failing.
12. Since starting medical school,	I feel unsupported by my peers.
13. Since starting medical school,	I feel competition from my peers.
14. Since starting medical school,	I feel unsupported by faculty.
15. Since starting medical school,	I feel taken advantage of by faculty (i.e. research mentors, professors, and/or school administrators).
16. Since starting medical school,	I feel pressure from others (i.e. parents, professors, mentors, etc.) to get good grades.
17. Since starting medical school,	I am overly self-critical.
18. Since starting medical school,	I feel unsure of my abilities as a student.
19. Since starting medical school,	I hardly have enough time to get things done.
20. Since starting medical school,	I feel overwhelmed by everything there is to do.
21. Since starting medical school,	I struggle maintaining a healthy school-life balance.
22. Since starting medical school,	I think about dropping out of school.

## Discussion

The purpose of this current study was to gather psychometric evidence for a new measure of medical student stress. The items comprising the MSSS are a distinctive mix of targeted and generic content that encapsulates the experience of stress in the medical school environment. The MSSS was developed using a rigorous, student-centered methodology that involved medical students, faculty, and experts in medical education, clinical psychology, and measurement development. Field-testing of the final item pool and external validity measures was conducted during required class time, so as not to burden the students’ busy schedules.

This study followed a well-established methodology of evaluating a scale’s dimensionality using multiple methods including exploratory item factor analysis and interpretability of resulting models by experts in the field. We then further refined the factor model using confirmatory item factor modeling, which showed that most of the potential multidimensionality was likely driven by item doublets and triplets, which may reflect local dependencies. Items were removed in these cases, to further meet the expectations for the third phase of quantitative modeling: IRT. However, multidimensionality in the MSSS remained, insofar as six items reflected social challenges experienced by medical students. This was modeled using a bi-factor IRT model. The final model had 22 items, of which 14 reflected only the general medical social stress and 8 also captured social challenges.

The MSSS is a flexible and precise measure of the different types and levels of stress commonly experienced by medical students. While the MSSS can discriminate very well between those who are experiencing medical student-related stress at varying levels of severity, some precision may decline among individuals experiencing very little stress, as well as for those at extreme levels. Additionally, the MSSS is able to detect statistically significant differences in stress levels between male and female medical students, as well as between first and second year medical students. In some situations, such as medical student year, the MSSS detected differences where other commonly used measures did not. Further, the MSSS demonstrated convergent validity evidence through high, significant associations with the existing measures of burnout, anxiety, and stress.

Our study was not without limitations. The sample was relatively small and drawn from just one institution. Additionally, all models were fit to the same dataset, including the exploratory use of CFA modeling, which limits the results. There were relatively fewer M3 and M4 students, who are known to experience comparatively higher levels of stress compared with M1s and M2s. Over 80% of participants were M1s and M2s, a discrepancy largely due to increased workload and minimal required group class time during third and fourth years to administer the survey. Additionally, given that the survey was optional for all participants, there is the possibility of a selection bias, with the extremes (either most or least stressed) students opting in or out. These factors may have implications on the generalizability of findings.

Future studies will benefit from confirming the proposed factor structure in a new sample, and evaluating its sensitivity to change over time. This is especially important considering possible uses of the MSSS include continuous screening and stress monitoring, as well as to evaluate the effectiveness of wellness interventions to reduce and manage stress in medical school. Since at this time no cut-points have been established to determine thresholds for mild, moderate, and severe levels of stress, future studies should also engage in standard setting activities to facilitate the clinical utility of this tool [21]. Lastly, when viewed on the basis of individual items, the MSSS may appear appropriate for any graduate student; however, when viewed in its 22-item totality, the MSSS represents the multifaceted elements of medical student stress and future testing alongside other student stress scales may help to elucidate further appropriateness for other student groups.

## Conclusion

The 22 items comprising the MSSS are an appropriate blend of specific and generic content relevant to medical student daily life and are supported by a high internal consistency and convergent /concurrent validity. The MSSS can provide accurate, precise, and relevant measurement of stress and burnout for the twenty-first century medical student. Future applications include use as an outcome measure in comparative effectiveness research or as a screening tool in an academic or clinical program.

### Practice points

- Medical students face unique and significant stressors
- Brief and precise measurement of medical student stress is paramount to identifying and ameliorating challenges
- Existing measures have limitations
- The MSSS-22 is a brief, IRT-derived measure with high relevance and precision
- The MSSS-22 performs as expected with legacy measures and discriminates well between specific groups

## Supplementary Information

**Additional file 1.** **Figure SF1**. Study Flow

**Additional file 2.** **Figure SF2**. Bifactor Model

**Additional file 3.** **Table ST1**. Marginal IRT Parameters with Plots for Item Characteristic Curves and Item Information Curves

**Additional file 4.** **Table ST2**. Item Response Theory-Derived T-Score Conversion Table

## Data Availability

The datasets used and/or analyzed during the current study are available from the corresponding author on reasonable request.

## Abbreviations

IRT: Item response theory
MSSS: Medical Student Stress Scale
PSS: Perceived Stress Scale
MBI: Maslach Burnout Inventory
PMSS: Perceived Medical Student Stress
MSSP: Medical Student Stress Profile
MSSQ: Student Stressor Questionnaire
PROMIS: Patient Reported Outcomes Measurement Information System®
VAS: Visual Analog Scale
EFA: Exploratory factor analysis
CFA: Confirmatory item factor analysis
REDCap: Research Electronic Data Capture
