# Supplementary figures and images for: Psychometric properties of a new self-report measure of medical student stress using classic and modern test theory approaches
Source: Health Qual Life Outcomes. 2021 Jan 2;19:2. doi: 10.1186/s12955-020-01637-0 (PMC7778790; doi:10.1186/s12955-020-01637-0)

**Supplementary Figure SF1. Study Flow**

**
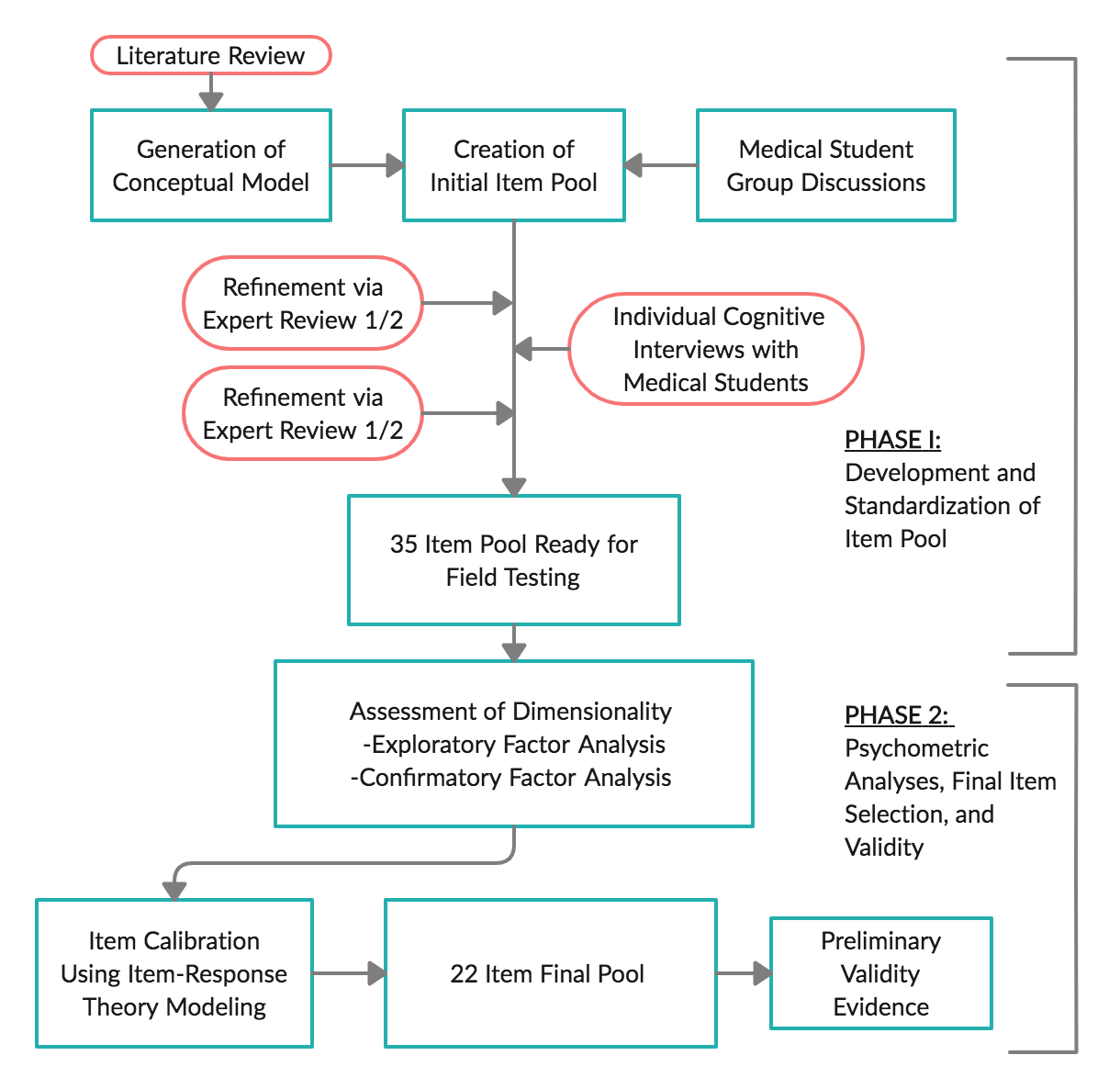
**

Supplement: Supplementary file 1 — Additional file 1. Figure SF1. Study Flow [file 12955_2020_1637_MOESM1_ESM.docx]

**Supplementary Figure SF2. Bifactor Model**


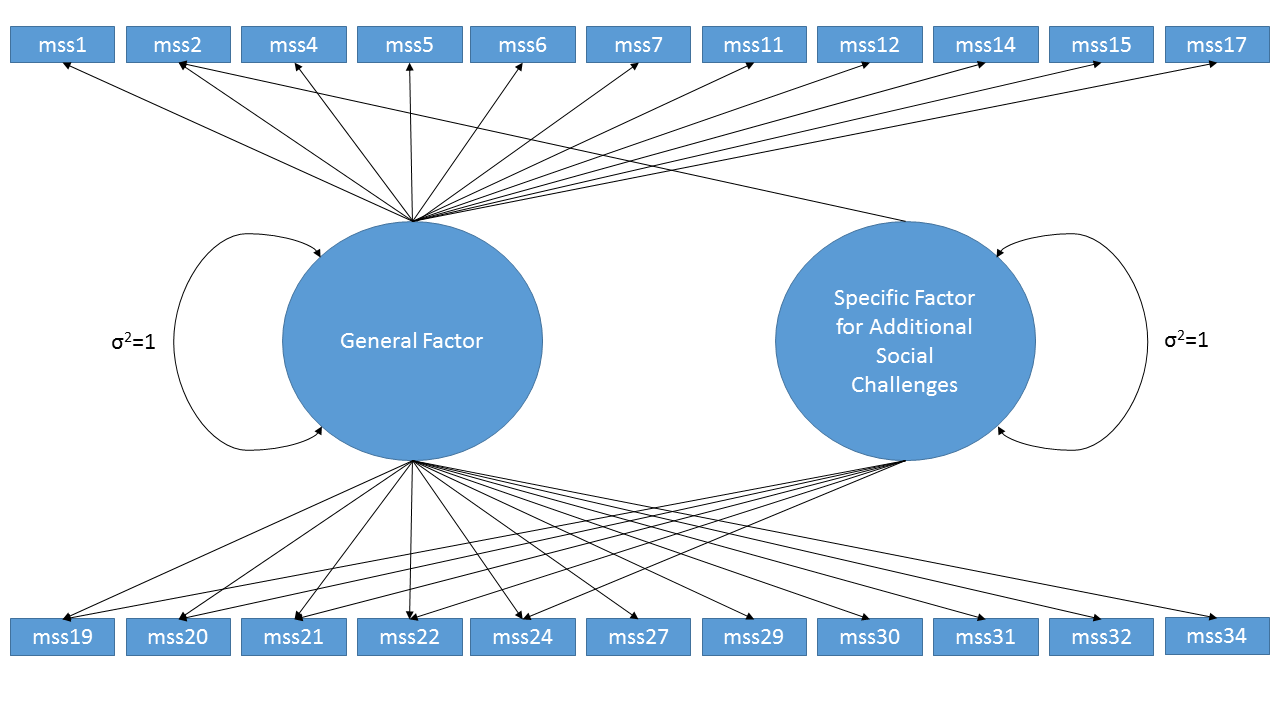

Supplement: Supplementary file 2 — Additional file 2. Figure SF2. Bifactor Model [file 12955_2020_1637_MOESM2_ESM.docx]
