# Supplementary material for: Psychometric properties of a new self-report measure of medical student stress using classic and modern test theory approaches
Source: Health Qual Life Outcomes. 2021 Jan 2;19:2. doi: 10.1186/s12955-020-01637-0 (PMC7778790; doi:10.1186/s12955-020-01637-0)
Supplement: Supplementary file 3 — Additional file 3. Table ST1. Marginal IRT Parameters with Plots for Item Characteristic Curves and Item Information Curves [file 12955_2020_1637_MOESM3_ESM.docx]

**Supplementary Table ST1. Marginal IRT Parameters with Plots for Item Characteristic Curves and Item Information Curves**

| **Item ID** | **Stem** | **General Factor Slope** | **Specific Factor Slope** | **Marginal Slope** | **Intercept / Marginal Threshold** | | | | **Average Threshold (Difficulty)** | **Item Characteristic Curve** |
| --- | --- | --- | --- | --- | --- | --- | --- | --- | --- | --- |
|  |  |  |  |  | **1** | **2** | **3** | **4** |  |  |
| MSSS30 | Not enough time to get things done | 1.24 |  | 1.24 | 5.54 | 2.71 | 0.57 | -1.51 | -1.476 | 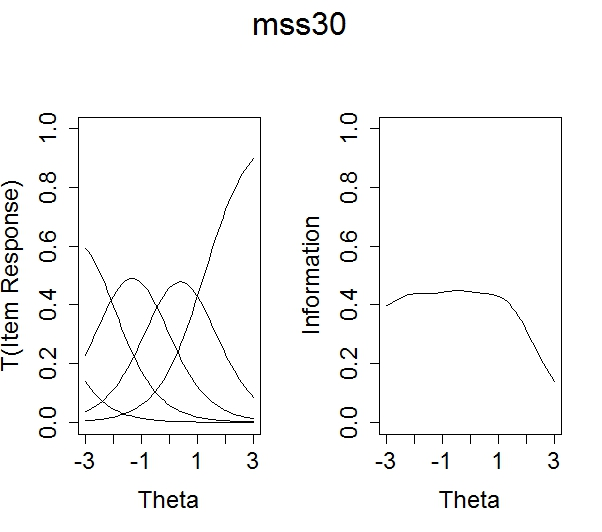 |
|  |  |  |  |  | -4.50 | -2.19 | -0.50 | 1.22 |  |  |
| MSSS27 | Overly self-critical | 1.54 |  | 1.54 | 5.36 | 2.50 | 0.03 | -1.78 | -0.991 | 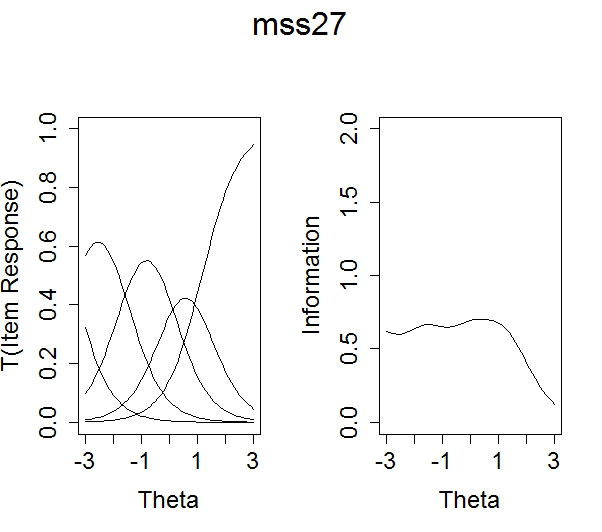 |
|  |  |  |  |  | -3.5 | -1.62 | -0.00 | 1.15 |  |  |
| MSSS31 | Overwhelmed by everything there is to do | 2.23 |  | 2.23 | 6.70 | 3.61 | 0.64 | -2.51 | -0.945 | 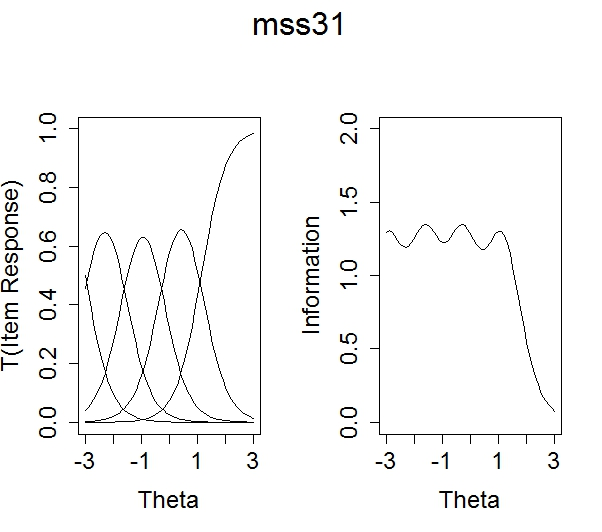 |
|  |  |  |  |  | -3.0 | -1.62 | -0.3 | 1.12 |  |  |
| MSSS6 | Feel anxious | 1.96 |  | 1.96 | 5.99 | 2.88 | -0.24 | -3.68 | -0.630 | 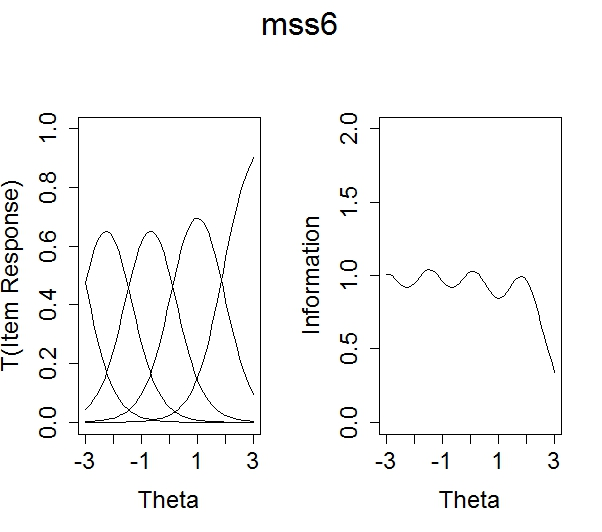 |
|  |  |  |  |  | -3.0 | -1.46 | 0.12 | 1.87 |  |  |
| MSSS32 | Struggle maintaining school-life balance | 1.72 |  | 1.72 | 5.03 | 2.19 | -0.44 | -2.88 | -0.565 | 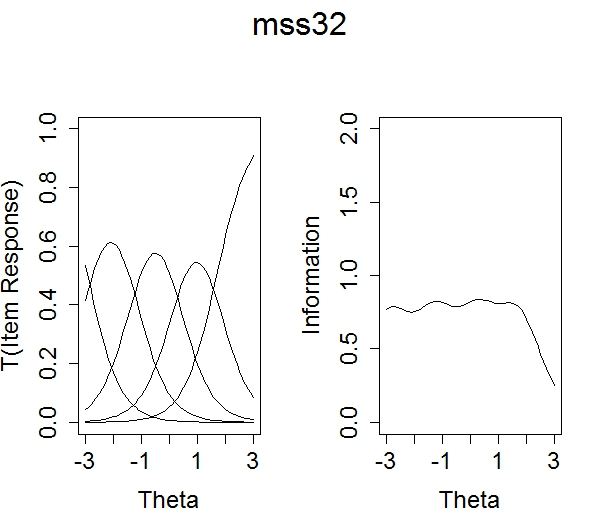 |
|  |  |  |  |  | -2.9 | -1.27 | 0.26 | 1.67 |  |  |
| MSSS29 | Unsure of abilities as student | 1.87 |  | 1.87 | 4.25 | 1.93 | -0.65 | -2.91 | -0.351 | 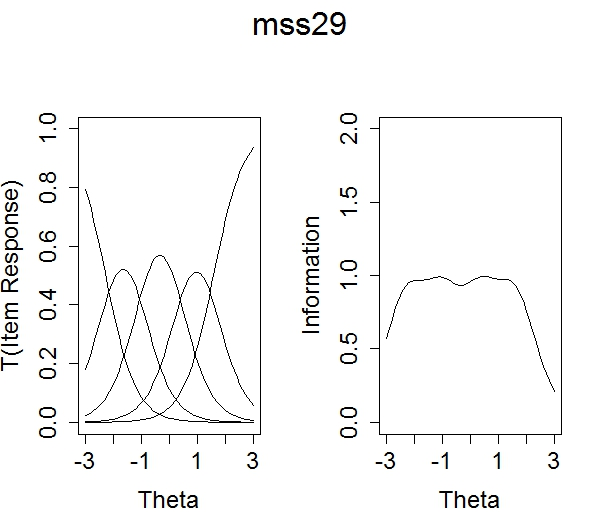 |
|  |  |  |  |  | -2.3 | -1.04 | 0.35 | 1.56 |  |  |

| MSSS15 | Emotionally exhausted | 1.91 |  | 1.91 | 4.13 | 1.75 | -0.89 | -3.57 | -0.185 | 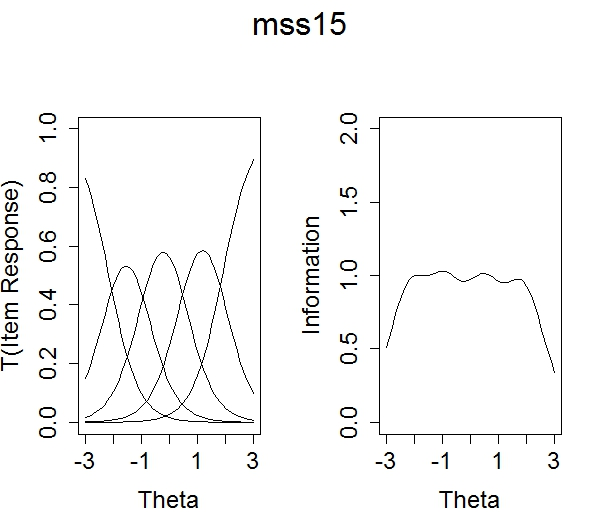 |
| --- | --- | --- | --- | --- | --- | --- | --- | --- | --- | --- |
|  |  |  |  |  | -2.20 | -0.92 | 0.47 | 1.87 |  |  |
| MSSS14 | Hard time motivating self to study | 1.08 |  | 1.08 | 3.37 | 1.46 | -1.04 | -3.28 | -0.116 | 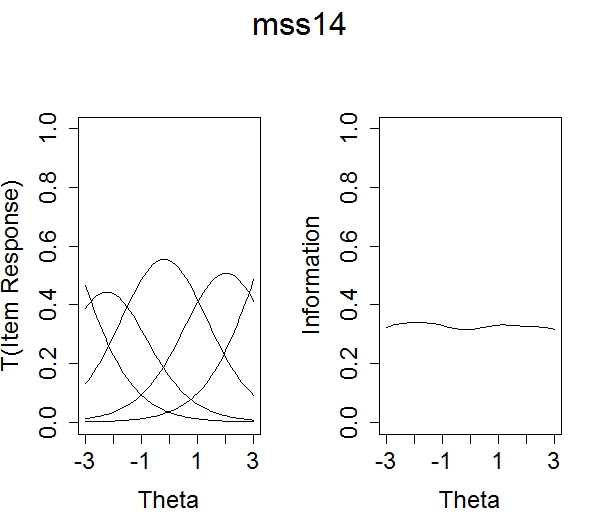 |
|  |  |  |  |  | -3.10 | -1.35 | 0.97 | 3.05 |  |  |
| MSSS5 | Unable to relax | 1.92 |  | 1.92 | 4.18 | 1.38 | -1.40 | -4.19 | 0.002 | 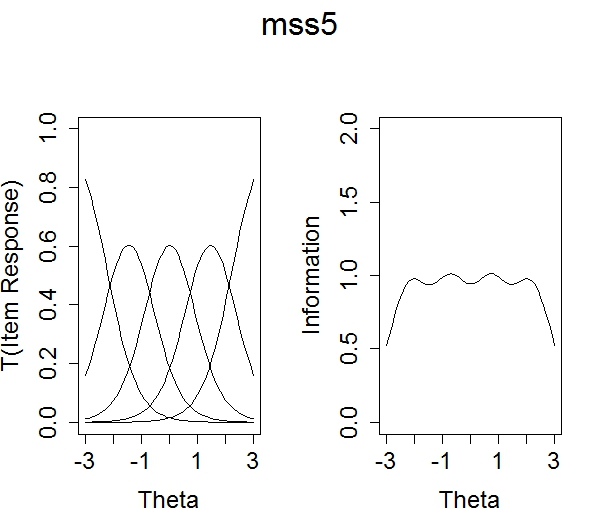 |
|  |  |  |  |  | -2.20 | -0.72 | 0.73 | 2.18 |  |  |

| MSSS17 | Fearful of failing | 1.96 |  | 1.96 | 3.09 | 1.02 | -1.28 | -3.78 | 0.121 | 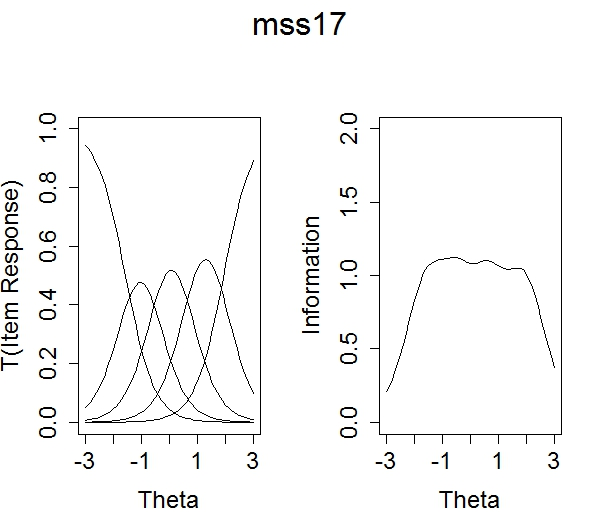 |
| --- | --- | --- | --- | --- | --- | --- | --- | --- | --- | --- |
|  |  |  |  |  | -1.60 | -0.52 | 0.65 | 1.92 |  |  |
| MSSS20 | Competition from peers | 0.82 | 0.91 | 0.72 | 3.54 | 0.75 | -1.40 | -3.74 | 0.258 | 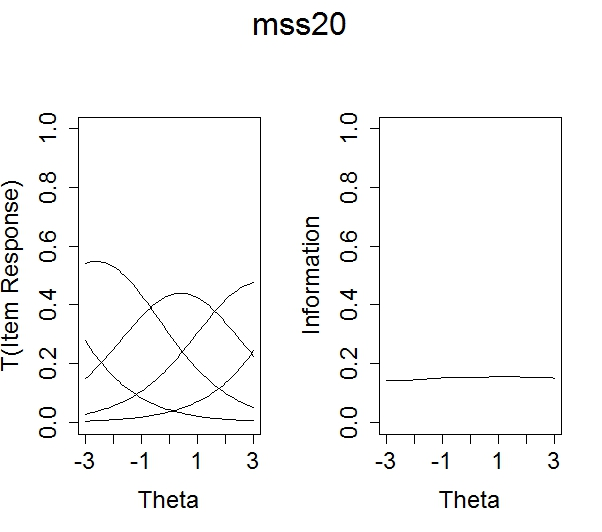 |
|  |  |  |  |  | -4.30 | -0.91 | 1.70 | 4.57 |  |  |
| MSSS1 | Fluctuations in appetite | 1.14 |  | 1.14 | 2.41 | 0.76 | -1.11 | -4.10 | 0.449 | 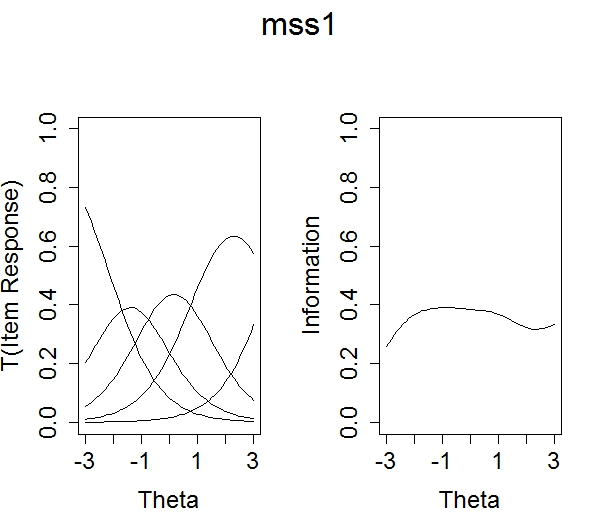 |
|  |  |  |  |  | -2.10 | -0.67 | 0.97 | 3.61 |  |  |

| MSSS4 | Less satisfaction from learning | 1.06 |  | 1.06 | 2.56 | 0.24 | -1.75 | -3.77 | 0.640 | 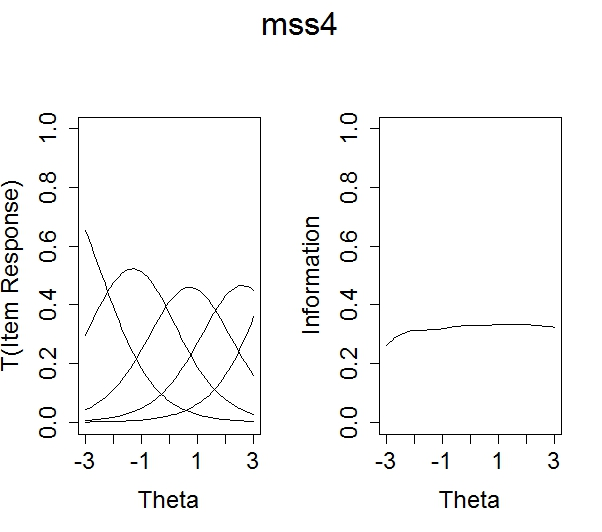 |
| --- | --- | --- | --- | --- | --- | --- | --- | --- | --- | --- |
|  |  |  |  |  | -2.40 | -0.22 | 1.65 | 3.54 |  |  |
| MSSS24 | Pressure from others to get good grades | 0.89 | 0.54 | 0.85 | 1.82 | 0.13 | -1.28 | -2.95 | 0.644 | 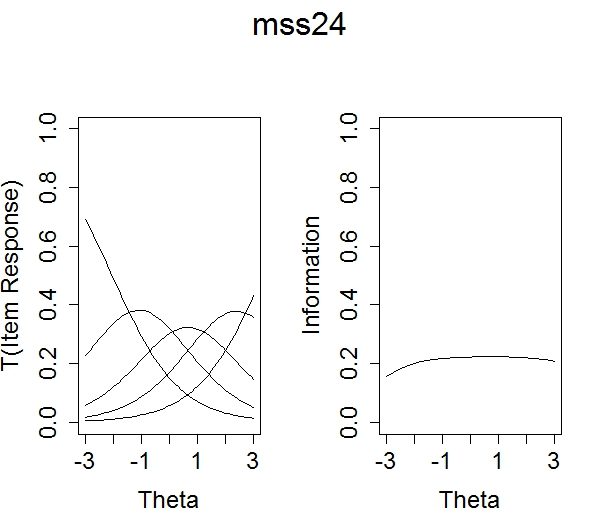 |
|  |  |  |  |  | -2.00 | -0.14 | 1.44 | 3.32 |  |  |
| MSSS2 | Difficulty asking for help | 1.37 | 0.78 | 1.24 | 3.10 | 0.30 | -2.60 | -5.19 | 0.801 | 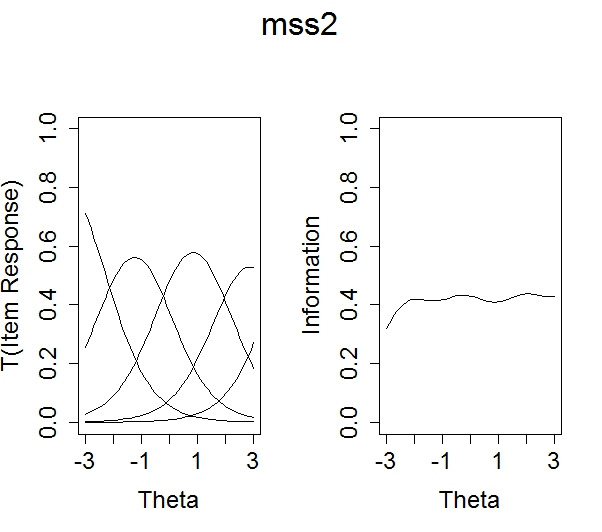 |
|  |  |  |  |  | -2.30 | -0.22 | 1.90 | 3.79 |  |  |

| MSSS12 | Feel depressed | 1.87 |  | 1.87 | 2.18 | -0.14 | -2.87 | -5.23 | 0.809 | 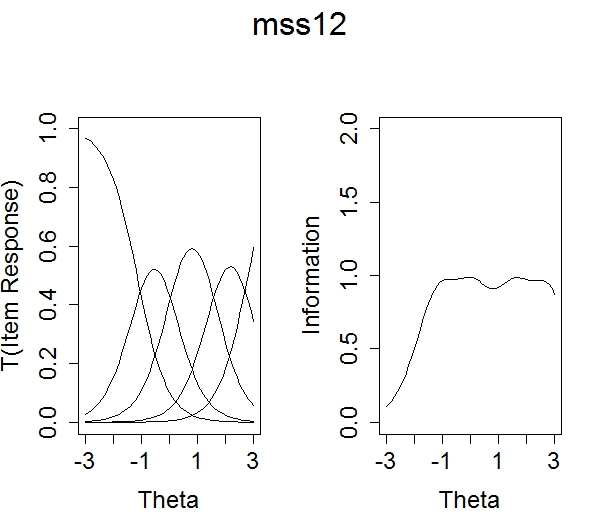 |
| --- | --- | --- | --- | --- | --- | --- | --- | --- | --- | --- |
|  |  |  |  |  | -1.20 | 0.07 | 1.53 | 2.79 |  |  |
| MSSS7 | Unable to enjoy activities outside of classes | 1.45 |  | 1.45 | 2.10 | -0.40 | -2.14 | -4.88 | 0.922 | 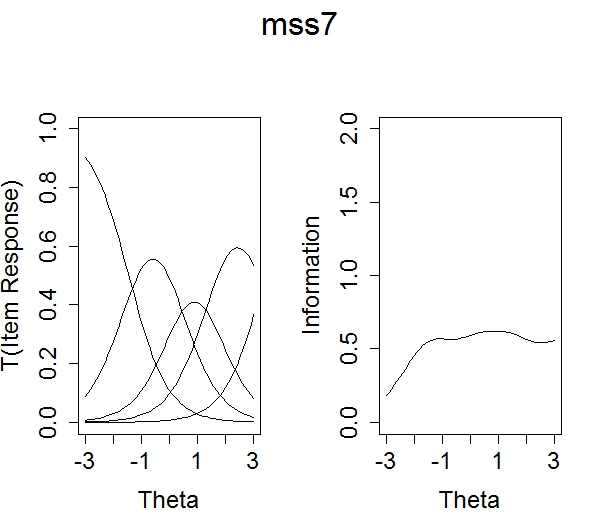 |
|  |  |  |  |  | -1.50 | 0.28 | 1.48 | 3.38 |  |  |
| MSSS21 | Unsupported by faculty | 1.47 | 2.02 | 0.94 | 3.09 | -0.54 | -3.39 | -7.24 | 1.38 | 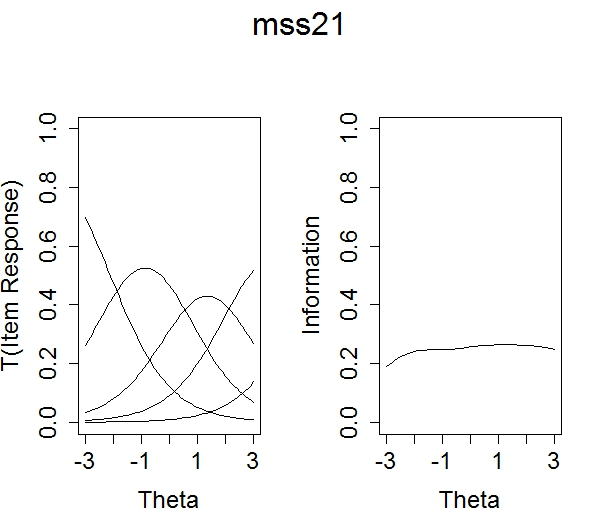 |
|  |  |  |  |  | -2.10 | 0.37 | 2.32 | 4.94 |  |  |

| MSSS19 | Unsupported by peers | 1.25 | 1.09 | 1.05 | 1.72 | -0.81 | -3.60 | -5.53 | 1.64 | 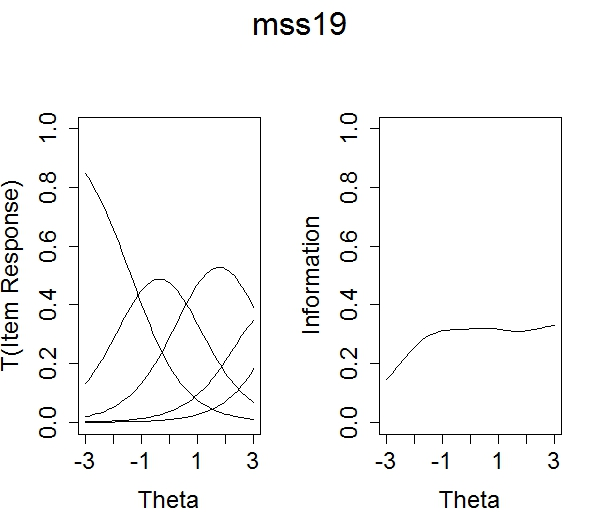 |
| --- | --- | --- | --- | --- | --- | --- | --- | --- | --- | --- |
|  |  |  |  |  | -1.40 | 0.651 | 2.88 | 4.42 |  |  |
| MSSS11 | Hopeless ever get degree | 1.82 |  | 1.82 | -0.07 | -2.27 | -4.06 | -6.45 | 1.76 | 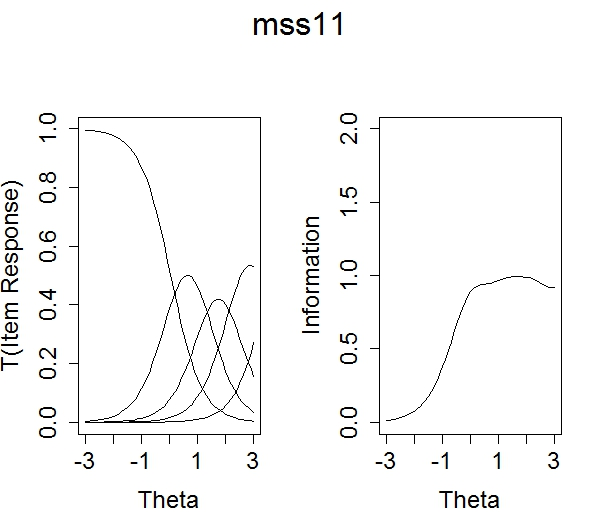 |
|  |  |  |  |  | 0.04 | 1.24 | 2.23 | 3.54 |  |  |
| MSSS34 | Think about dropping out | 1.38 |  | 1.38 | -0.08 | -1.65 | -3.09 | -5.40 | 1.85 | 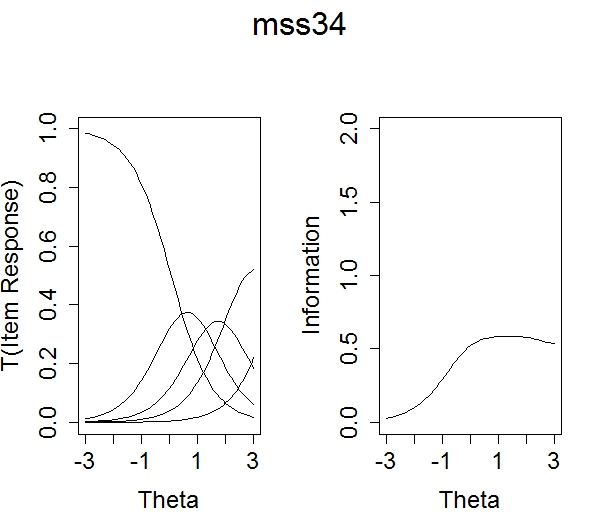 |
|  |  |  |  |  | 0.06 | 1.20 | 2.24 | 3.92 |  |  |

| MSSS22 | Feel taken advantage of by faculty | 0.68 | 1.34 | 0.54 | 0.21 | -1.92 | -3.80 | -5.27 | 3.94 | 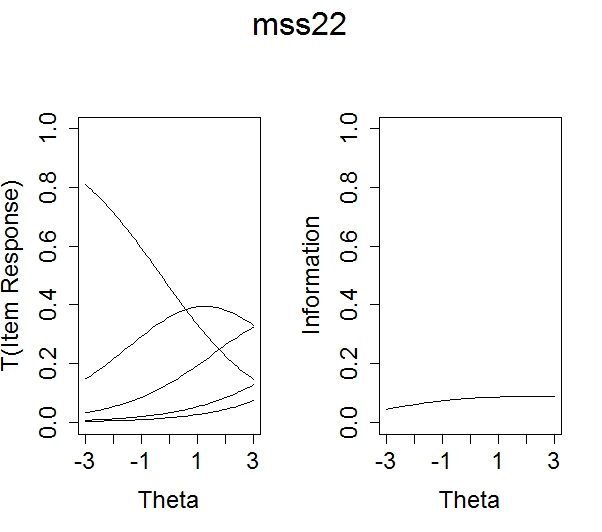 |
| --- | --- | --- | --- | --- | --- | --- | --- | --- | --- | --- |
|  |  |  |  |  | -0.30 | 2.81 | 5.55 | 7.71 |  |  |

Note: *This presents both the bifactor parameters in slope-intercept format, with marginal slopes on the general factor and marginal thresholds, for the multidimensional elements.*
