# Supplementary material for: Psychometric properties of a new self-report measure of medical student stress using classic and modern test theory approaches
Source: Health Qual Life Outcomes. 2021 Jan 2;19:2. doi: 10.1186/s12955-020-01637-0 (PMC7778790; doi:10.1186/s12955-020-01637-0)
Supplement: Supplementary file 4 — Additional file 4. Table ST2. Item Response Theory-Derived T-Score Conversion Table [file 12955_2020_1637_MOESM4_ESM.docx]

| **Supplementary Table ST2.**  **Item Response Theory-Derived T-Score Conversion Table** | | |
| --- | --- | --- |
| MSSS Total  Raw Score | T-Score | Standard Deviation |
| 0 | 10.37 | 4.72 |
| 1 | 12.66 | 4.41 |
| 2 | 14.69 | 4.19 |
| 3 | 16.53 | 4.03 |
| 4 | 18.20 | 3.90 |
| 5 | 19.74 | 3.80 |
| 6 | 21.17 | 3.71 |
| 7 | 22.50 | 3.65 |
| 8 | 23.76 | 3.59 |
| 9 | 24.94 | 3.54 |
| 10 | 26.07 | 3.50 |
| 11 | 27.15 | 3.46 |
| 12 | 28.19 | 3.43 |
| 13 | 29.19 | 3.41 |
| 14 | 30.15 | 3.38 |
| 15 | 31.10 | 3.37 |
| 16 | 32.01 | 3.35 |
| 17 | 32.91 | 3.34 |
| 18 | 33.79 | 3.32 |
| 19 | 34.65 | 3.32 |
| 20 | 35.49 | 3.31 |
| 21 | 36.32 | 3.30 |
| 22 | 37.14 | 3.30 |
| 23 | 37.95 | 3.29 |
| 24 | 38.74 | 3.29 |
| 25 | 39.53 | 3.29 |
| 26 | 40.30 | 3.28 |
| 27 | 41.07 | 3.28 |
| 28 | 41.83 | 3.28 |
| 29 | 42.58 | 3.28 |
| 30 | 43.32 | 3.28 |
| 31 | 44.06 | 3.28 |
| 32 | 44.79 | 3.28 |
| 33 | 45.51 | 3.28 |
| 34 | 46.23 | 3.28 |
| 35 | 46.95 | 3.28 |
| 36 | 47.66 | 3.28 |
| 37 | 48.37 | 3.28 |
| 38 | 49.08 | 3.28 |
| 39 | 49.78 | 3.28 |
| 40 | 50.48 | 3.28 |
| 41 | 51.18 | 3.28 |
| 42 | 51.87 | 3.28 |
| 43 | 52.57 | 3.29 |
| 44 | 53.26 | 3.29 |
| 45 | 53.95 | 3.29 |
| 46 | 54.64 | 3.30 |
| 47 | 55.33 | 3.30 |
| 48 | 56.01 | 3.30 |
| 49 | 56.70 | 3.31 |
| 50 | 57.39 | 3.32 |
| 51 | 58.08 | 3.32 |
| 52 | 58.78 | 3.33 |
| 53 | 59.47 | 3.34 |
| 54 | 60.17 | 3.35 |
| 55 | 60.86 | 3.36 |
| 56 | 61.57 | 3.38 |
| 57 | 62.27 | 3.39 |
| 58 | 62.99 | 3.41 |
| 59 | 63.70 | 3.42 |
| 60 | 64.42 | 3.44 |
| 61 | 65.15 | 3.47 |
| 62 | 65.89 | 3.49 |
| 63 | 66.63 | 3.52 |
| 64 | 67.38 | 3.55 |
| 65 | 68.14 | 3.58 |
| 66 | 68.90 | 3.62 |
| 67 | 69.68 | 3.65 |
| 68 | 70.47 | 3.69 |
| 69 | 71.26 | 3.74 |
| 70 | 72.07 | 3.78 |
| 71 | 72.89 | 3.83 |
| 72 | 73.72 | 3.88 |
| 73 | 74.56 | 3.94 |
| 74 | 75.42 | 4.00 |
| 75 | 76.30 | 4.06 |
| 76 | 77.19 | 4.12 |
| 77 | 78.11 | 4.18 |
| 78 | 79.04 | 4.25 |
| 79 | 80.01 | 4.31 |
| 80 | 81.02 | 4.37 |
| 81 | 82.06 | 4.44 |
| 82 | 83.16 | 4.49 |
| 83 | 84.32 | 4.55 |
| 84 | 85.57 | 4.60 |
| 85 | 86.92 | 4.65 |
| 86 | 88.42 | 4.70 |
| 87 | 90.11 | 4.77 |
| 88 | 92.13 | 4.90 |
